# Families created via identity-release egg donation: disclosure and an exploration of donor threat in early childhood

**DOI:** 10.1016/j.rbmo.2023.05.007

**Published:** 2023-05-13

**Authors:** Joanna Lysons, Susan Imrie, Vasanti Jadva, Susan Golombok

**Affiliations:** 1 Centre for Family Research, University of Cambridge, Cambridge, UK; 2 Institute for Women’s Health, Faculty of Population Health Sciences, University College London, London, UK

**Keywords:** Disclosure, Donor threat, Egg donation, Gamete donation, Identity release, IVF

## Abstract

**Research question:**

What are mothers’ disclosure intentions and practices from infancy to early childhood, and is perceived donor threat associated with disclosure in identity-release egg donation families when the children are aged 5 years?

**Design:**

This longitudinal study included 73 heterosexual-couple families with infants born following IVF-egg donation at phase one, and 61 families with 5-year-old children at phase two. At both phases, mothers were interviewed about their disclosure intentions and practices. At phase two, mothers were interviewed about their feelings about future donor–child contact.

**Results:**

Most mothers (75.3%) intended to disclose their use of egg donation to their children at phase one; half had begun to do so when their children were aged 5. Most remaining mothers planned to tell, although a minority were uncertain or planned not to disclose. When the child was aged 5, four mothers had started telling them that they could access their donor’s identifying information at age 18, and most (84%) intended to do so in the future. Most couples agreed on a disclosure strategy at phase two. Most mothers perceived at least some threat from future donor–child contact, but this was unrelated to their disclosure practices.

**Conclusions:**

Disclosure intentions in infancy are borne out in early childhood. Despite perceiving some threat from future donor – child contact, most mothers intended telling their child that they could access the donor’s identifying information at age 18. Revisiting these families as the children grow older will be important to understand how the mothers’ perceived donor threat may change over time, and how this is related to family processes.

## Introduction

**S**ince 2005 and the removal of donor anonymity, identity-release donation has been the main option available to individuals seeking fertility treatment with donor eggs in the UK. This means that patients do not know the donor*’*s identity at the time of treatment, but any resultant child has the right to access identifying information about the donor (i.e. their full name, date of birth and last known address) from age 18 years. Over 4000 treatment cycles involving donor eggs were carried out in the UK in 2018 (Human Fertilisation and Embryology Authority, 2020). Identity-release donation is also the main treatment option for individuals requiring treatment with donor eggs in several countries internationally, including Sweden, Norway, New Zealand and Australia. In other countries, such as the USA and Denmark, patients may choose whether to pursue egg donation with an anonymous or an identifiable donor. Despite the growing use of identity-release egg donation, little is known about the outcomes for families created through this technology (Imrie and Golombok, 2018).

Parents conceiving via donor eggs must decide whether or not they intend to tell their child about their donor conception. In many high-income countries, including the UK, there has been a trend over the last two decades towards encouraging parents to disclose donor conception to their children (Collins, 2022; *Donor Conception Network, n.d*.; HFEA, 2021; *Nuffield Council of Bioethics, 2013*). In the UK, the current Human Fertilisation and Embryology Authority Code of Practice states that clinics must give patients information about *‘*the importance of telling any resultant children, at an early age, of their donor-conceived origins*’* (HFEA, 2021a, paragraph 20.6 –20.7). Similarly, the *Ethics Committee of the American Society of Reproductive Medicine (2018)* strongly encourages parents to inform their children of their donor conception, although it does also state that the decision of whether or not to disclose donor conception is a parent*’*s choice, given the highly personal nature of the decision (ASRM, 2018).

Disclosure rates amongst cisheterosexual couples with families created through egg donation vary between studies, with most samples comprising parents who used anonymous donation (i.e. when the donor*’*s identity will never be known). The only longitudinal study of UK egg donation families (of whom the majority had used anonymous donation) found that, when they were interviewed during their child*’*s infancy, 56% of heterosexual-couple egg donation parents intended to disclose, and that by the time the child was 7 years of age, 41% had done so (Blake et al., 2014). A survey of 167 Finnish families created through anonymous or known egg donation found that those with younger children were more likely to report intention to disclose than those with older children (Soederstroem-Anttila et al., 2010), suggesting that changing attitudes towards disclosure may also be seen among egg donation parents. Whether this is also the case in other cultural contexts is not known.

It is unknown how identity-release legislation may impact parents*’* disclosure intentions. It has been suggested that identity release may add an additional level of complexity to an already complex process, even potentially leading to greater levels of secrecy rather than openness (English et al., 2002; Freeman et al., 2016). Only two studies have addressed this question directly. Isaksson and colleagues found that, of 55 Swedish identity-release egg donation families with 1-to 4-year-olds, 18% of couples had already disclosed the egg donation to the child and 75% intended to tell them (Isaksson et al., 2012). A follow-up study found that 61% of families had disclosed by the time their child was aged 7–8 years (Lampic et al., 2021).

Despite identity-release donation being the most common form of egg donation treatment in the UK since 2005, nothing is known about UK parents*’* disclosure intentions in families created using identity-release donation. With the first UK cohort of children conceived via identity-release donation turning 18 now, in 2023, understanding parents*’* attitudes towards the disclosure of identity-release donation is particularly pertinent. The present paper therefore uses findings from two phases of a longitudinal study of UK families with children conceived via identity-release egg donation, to answer the following research question: what are mothers*’* disclosure intentions and practices with regards to disclosure to their children from infancy to early childhood?

There is also the issue of whether knowing the donor is felt to be a threat. A small but growing body of literature suggests that the parents of children conceived via identity-release gamete donation feel to some degree threatened by the prospect of donor–child contact in the future. Widbom and colleagues found that, among 23 families with adult children conceived via identity-release sperm donation, the fathers demonstrated discomfort with the idea of their child obtaining information about their donor, with one father describing the prospect as *‘*something sinister and dark … and threatening to the fatherhood and to the role of the male in the family*’* (Widbom et al., 2021). Similarly, some parents in Isaksson and co-workers*’* study of 30 families with 7-year-old children conceived via identity-release sperm donation reported concerns about future donor–child contact and about what kind of person the donor might be (Isaksson et al., 2016).

As part of the present study, Lysons and colleagues (2022) found that although some mothers of children conceived via identity-release egg donation viewed future donor–child contact as an exciting opportunity, many viewed it as a threat to their identity as mothers and to the mother –child relationship. This study also found that, for some mothers, their fears were compounded by the fact that, because identity-release is a relatively new system in the UK, there is a dearth of information about what donor–child contact might look like for donor conceived children and their parents.

It has been suggested that the perceived threat posed by future donor–child contact may put pressure on the parent –child relationship (Lampic et al., 2014) and that perceived donor threat may make parents less likely to tell their child about their method of conception (Imrie et al., 2020). The literature on disclosure among children conceived with anonymous donors provides some subtle evidence of perceived threat to the parent –child relationship: while all the parents in a sample of 19 heterosexual-couple surrogacy parents who had used a genetic surrogate had disclosed their use of surrogacy by the time their child was 10 years old, only 58% had disclosed that they had used the surrogate*’*s egg (Jadva et al., 2012).

A study of mothers single by choice and heterosexual partnered mothers who had used sperm donation found that fewer partnered mothers than single mothers had disclosed their use of donor conception to their child (Freeman et al., 2016). Among the participants in that study who had not disclosed, partnered mothers were significantly more negative about disclosure than single mothers. Similarly, a study of single mothers and lesbian couple and heterosexual couple parents found that although rates of disclosure were relatively high across all groups, heterosexual couple parents were significantly less likely to disclose their use of sperm donation to their child (Scheib et al., 2003).

Together these findings indicate that, among cisheterosexual coupled parents where one parent lacks a genetic link with the child, the donor may be perceived as somewhat threatening. However, no study has yet attempted to overtly quantify levels of perceived threat posed by an identifiable egg donor, nor has any empirical work examined this in relation to mothers*’* disclosure practices. The present study therefore also aims to answer a second research question: does perceived donor threat relate to mothers*’* disclosure to their children in identity-release egg donation families when the children are aged 5 years?

## Materials and Methods

### Participants

The sample forms part of a larger longitudinal study examining family functioning in families created through fertility treatment. At phase one, heterosexual-couple families who had had privately funded fertility treatment and had had a child in the previous 3 –12 months were recruited through 12 UK fertility clinics. In order to maintain confidentiality, all the families were contacted by the clinics in the first instance, and were invited to submit their contact details to the research team in order to register their interest in the study. Clinics contacted a total of 419 families, of which 190 submitted contact details to the research team; the overall participation rate at phase one was 87% (full details of the recruitment procedure are provided in Imrie et al., 2019a). Families gave their consent to be contacted by the research team in the future and were subsequently contacted by a member of the research team at phase two, shortly before the target child*’*s 5th birthday. The overall retention rate between phases one and two was 85%.

Seventy-three families who had conceived through identity-release egg donation in the UK participated in the study at phase one. The mothers were aged 33 –52 years (mean 42.71 years, SD = 4.08) and the fathers were aged 32 – 62 years (mean 43.90 years, SD = 6.63). Families had infants aged 6 –18 months (mean 11.26 years, SD = 2.10). Sixty-one families participated again at phase two; the mothers were aged 38 –57 years (mean 47.30 years, SD = 4.37) and the fathers were aged 37 – 67 years (mean 48.6 years, SD = 6.42). The children were aged 5 years at the time of the visit (mean 67.5 months, SD = 4.08).

An age of 5 years was selected as the target age at phase two as this is roughly the age by which clinics and support groups advise parents to have begun the disclosure process (*Donor Conception Network, n.d*.; HFEA, 2021). Moreover, children*’*s transition to school at age 5 coincides with developments in their social understanding (Hughes, 2011) and in their understanding of genetic relatedness and heritability, which is thought to develop between the ages of 5 and 7 (Brodzinsky, 2011; Solomon et al., 1996; Williams and Smith, 2010). Early childhood may therefore represent a period during which identity-release egg donation parents begin to feel an increasing expectation to begin the disclosure process, and this may, in turn, catalyse thoughts about the donor and the possibility of future donor–child contact.

Mothers and fathers were interviewed as part of the larger study (see Imrie et al., 2019a, 2019b; Jadva et al., 2022; Lysons et al., 2022). All the mothers identified their ethnicity as White British. The majority of mothers (70%) and fathers (69%) had a higher education qualification and were relatively wealthy, with 35% of mothers and 58% of fathers earning an above-average annual wage (>£33,000; *Office for National Statistics, 2022*). All the mothers were either married or in non-marital cohabiting relationships at phase one; at phase two, the majority (93%) of couples remained in intact relationships.

### Procedure

At both phases of the study, the families were visited at home by one of two trained researchers. Written informed consent was obtained from both parents. Parents were administered a semi-structured interview that was audio-recorded and later transcribed verbatim; mothers and fathers were interviewed separately. Data were collected between October 2013 and June 2015 at phase one, and between July 2018 and December 2019 at phase two. Ethical approval was granted by the University of Cambridge Ethics Committee on 11 July 2013 (reference: PRE.2013.61) and 12 June 2018 (reference: PRE.2018.047).

As interview data were available for more mothers than fathers, and as mothers were the parent in this sample who lacked a genetic relationship with their child, data regarding disclosure and donor threat are reported from mothers*’* interviews. Where data regarding disclosure were available for both members of the couple, the level of agreement on disclosure between mothers and fathers was calculated.

### Materials

#### Disclosure

At phase one, parents were asked whether or not they intended to tell their child about their donor conception, and their responses were coded according to the three categories of *plans not to tell, uncertain* and *plans to tell*. Participants who planned to tell were asked about the age at which they planned to tell their child. At phase two, parents were again asked whether they had told their child, or intended to tell their child, about their donor conception, and their responses were coded into the categories above but with an additional fourth category, *started telling*.

In addition, parents were also asked whether they had told their child that they would be able to access the donor*’*s identifying information in the future. Parents*’* responses were coded into the four categories above. As detailed in Lysons and colleagues (Lysons et al., 2022), almost one-third of parents in this sample (28% of mothers, 31% of fathers) did not fully understand that they had used an identifiable donor. Disclosure at phase two was therefore also analysed by the mothers*’* level of understanding about identity-release.

#### Donor threat

Qualitative content analysis was conducted to develop a variable that captured the extent to which egg donation mothers viewed identity-release egg donation as a threat. Specifically, this variable was created to assess egg donation mothers*’* perceived threat from identity-release egg donation, and the potential for future donor–child contact. Codes were developed drawing from examples from the adoption literature that attempt to capture the variance in adoptive parents*’* feelings around confidentiality versus openness in adoption (see Grotevant et al., 1994).

Interview material coded for this variable was specific to mothers*’* thoughts and feelings about the prospect of donor–child contact, and included statements about fear of rejection from the child specifically in favour of the donor, fear of the donor claiming the child as their own, and fear of the donor – child bond being more legitimate than the mother –child bond. Mothers were rated as perceiving (a) *no threat*, (b) *little threat*, (c) *moderate threat*, or (d) *high threat*.A code book was produced, providing detailed instructions for coding including examples of content for each level of the variable. To establish inter-rater reliability, two-thirds of the mothers*’* transcripts were coded by a second rater. The intra-class coefficient was 0.84, indicating excellent reliability.

#### Donor threat and disclosure

A point-biserial correlation was conducted in order to examine whether a relationship existed between perceived donor threat and disclosure status at phase two. In order to create a binary disclosure variable, disclosure status was recoded so that *started telling* was recoded as *disclosed* (*n* = 27) and *plans to tell* was recoded as *not disclosed (n* = 17).

## Results

### Disclosure to the child

TABLE 1 summarizes the mothers*’* disclosure intentions at phase one and disclosure practices at phase two. At phase one, when the children were infants, 55 mothers (75.3%) planned to tell their child about their method of conception. Twelve mothers (16.4%) were uncertain, and the remainder of mothers (*n* = 6, 8.2%) planned not to disclose their use of identity-release egg donation to their child. At phase two, when the children were aged 5 years, 31 mothers (50.8%) had begun the disclosure process. A further 22 (36.1%) mothers planned to tell their child about their method of conception, while a minority of mothers (*n* = 4, 6.6%) were uncertain. Four mothers (6.6%) intended not to tell their child about their method of conception.

At phase two, of the mothers who had not yet disclosed but planned to tell, the majority (*n* = 9, 40.9%) planned to do so by the time their child reached 7 years old. Four mothers (18.2%) intended to tell between the ages of 7 and 10, while three mothers (13.6%) planned to disclose at some point during their child*’*s teens. The remaining six (27.3%) mothers who planned to disclose to their child were unsure of when they would do so (TABLE 2).

Of the families for whom data were available at both phases, 51 mothers at phase one had planned to tell. Thirty (58.8%) of these had started telling as planned. Eighteen mothers (35.3%) still planned on, but had not yet begun, telling. At phase two the remaining three mothers who had planned at phase one to tell were either uncertain (*n* = 2) or planned not to tell (*n* = 1). Of the six mothers who were uncertain about disclosure at phase one, four at phase two planned to tell, while two remained uncertain. Finally, three of the four mothers who had at phase one planned not to disclose still planned not to disclose at phase two; the remaining mother responded at phase two that she was uncertain whether to disclose.

### Couple agreement about disclosure at phase two

TABLE 3 presents the levels of agreement between couples at phase two. Mother and father data were available for 48 couples. The majority (*n* = 37, 77.1%) of couples agreed upon their disclosure strategy at phase two; however, a minority (*n* = 11, 22.9%) had mismatched disclosure intentions. The most common mismatch was where mothers had started the disclosure process and the corresponding fathers said that they planned to, but had not yet begun to, disclose (*n* = 4). In three couples, mothers said that they planned to tell, whereas the corresponding fathers indicated that they had started telling. Two couples had mothers who were uncertain about telling with corresponding fathers who planned not to tell, and one couple had a mother who planned to tell and father who planned not to tell. Finally, the inverse was true for one couple, such that the father planned to tell and the corresponding mother did not.

### Disclosure by level of understanding of identity release

TABLE 1 summarizes mothers*’* disclosure practices by level of understanding about identity release. Seventeen mothers did not understand that they had used identity-release egg donation; of these, four mothers had already begun the disclosure process. A further five planned to tell their child about their method of conception, and four mothers in this group were uncertain whether they would disclose their use of egg donation to their child. Four mothers in this group planned not to disclose. Of the 44 mothers who understood that they had used an identity-release egg donor, 27 (61.4%) had begun the disclosure process by the time their child was 5 years old. All the remaining mothers in this group planned to tell their child about their method of conception.

### Disclosure of identity-release

Among the 44 mothers who understood the principles of identity-release donation, four mothers (9.1%) had told their child that they would be able to access the donor*’*s identifying information in the future. Most (*n* = 37, 84.1%) of the remaining mothers planned to tell their child about identity release in the future, while a minority (*n* = 3, 6.8%) remained uncertain. The four mothers who had begun explaining identity-release donation to their child generally did so by sharing a basic level of information about the donor, and then telling their child that they would be able to find out more about the donor when they were older. Some mothers, like Sofia, did so in response to their child*’*s questions about the donor:

She has asked about her, and I*’*ve said I don*’*t know very much at all but when she*’*s older she can find out more about her, and that I know what colour eyes she*’*s got, how tall she is and what colour hair she*’*s got.

One mother, slightly further along in the disclosure process, had begun adding detail to her discussion of identity-release donation by seeding the concept of same-donor offspring:

I was talking to them about it this morning and I was saying, *‘*well, the kind ladies, one day you*’*re going to be able to meet your kind ladies and your kind ladies have also had children.*’* So … it*’*s the first time I sort of said, *‘*Oh, you*’*ve got half-sisters or half-brothers out there that you might meet when you*’*re older.*’* (Henrietta)

### Donor threat

Qualitative content analysis was conducted with the subsample of mothers who understood they had used identity-release donation (*n* = 44) in order to ascertain the extent to which identity-release donation was perceived as threatening. The majority (*n* = 20, 45%) of mothers were coded as perceiving *no threat* from identity-release egg donation; these mothers demonstrated an ability to conceptually coexist with the donor without any difficulty or residual fear, appeared comfortable with future donor – child contact, and demonstrated either neutrality or warmth when talking about the prospect, like Hannah:

I*’*m expecting [child] to want to contact the donor because I would. This is really weird, but we*’*d be disappointed if she doesn*’*t, because I can*’*t make that decision, but I would like to meet the person!

A total of 32% (*n* = 14) of mothers were coded as perceiving *little threat* from identity-release egg donation. This code meant that mothers were generally positive about identity-release egg donation but expressed a small amount of uncertainty or hesitation about the prospect of future donor – child contact, as demonstrated by Gabby:

I think in an ideal world maybe you wouldn*’*t ever want to tell them because you wouldn*’*t want anything to come between you or what have you. But then I always think about, you know, a lot of women were getting egg donation in [country] and were going there specifically because [country*’*s] law keeps the details of donors anonymous and then I*’*m just thinking you couldn*’*t do that to a child, you know, that*’*s part of them, but they*’*re never ever to know or never ever to find out? That must be really difficult, you know? So, I think it*’*s important that they do know.

A minority (*n* = 6, 14%) of mothers were coded as perceiving a *moderate threat* from identity-release egg donation; mothers coded at this level displayed marked ambivalence about identity-release donation and typically repeated one or two fears about identity release throughout the interview, while still making attempts to rationalize or reconcile their feelings with the child*’*s right to access identifying information about the donor:

I think I wouldn*’*t want her to [access the donor*’*s information] because I think I*’*d want her to just think that*’*s how it was and that*’*s it .. . but I think as an adult I know, because we*’*ve been gifted with that opportunity, then if she wants to do that that would have to be her choice, as much as I don*’*t think I .. . probably .. . realistically, I probably don*’*t think I want her to but I won*’*t stop her from doing it. (Hermione)

A small proportion of mothers (*n* = 4, 9%) were coded as perceiving *high threat* from identity-release egg donation. Mothers coded as perceiving *high threat* expressed pervasive fear about the prospect of future donor–child contact, and repeatedly referenced multiple different concerns about identity-release donation. Typically, the mothers did not wish to disclose their use of identity-release egg donation because of these fears, or worried about their decision to disclose because of their fears about identity release, like Martha:

I know she*’*s entitled to [the donor*’*s information], and it*’*s splashed all over her notes so she*’*s going to find out, but if there was any way of her not finding out I would do that. I would do anything for her not to find that out.

In one case, a mother*’*s decision not to disclose was specifically due to fear that her children would reject her in favour of the donor when they were old enough to access identifying information.

### Donor threat and disclosure

A point-biserial correlation was conducted to examine whether a relationship existed between donor threat and disclosure practices at phase two. No association was found between the two variables (r_pb_ = –0.002, *P* = 0.98).

## Discussion

The present study found that, when their children were in their infancy, the majority of mothers intended to disclose their use of egg donation to their children, and of these, just over half had begun to do so when their children were aged 5 years. Most mothers who had not disclosed by the time their child was aged 5 intended to do so in future, with only a minority of mothers intending not to disclose. The majority of the mothers who were unsure about disclosure at phase one had changed their minds at phase two and instead intended to tell their child, but had not yet done so. The few mothers who planned not to disclose at phase one remained consistent in their intentions at phase two. Of the mothers who understood they had used an identifiable donor, a handful had begun telling their child that they could access the donor*’*s identity in the future, with the remainder intending to disclose this detail to their child in the future. The present study is thus the first to report the disclosure intentions and practices of mothers with children conceived via identity-release egg donation in the UK.

Although some studies of anonymous donation have found that parents*’* disclosure intentions in infancy do not necessarily match disclosure practices later in life, the present findings suggest that, generally, mothers*’* disclosure intentions in infancy are borne out in early childhood and suggest that, rather than reducing disclosure rates, the removal of donor anonymity is concurrent with a continuing global trend towards openness among families undergoing assisted reproductive technology (ASRM Ethics Committee, 2018; Isaksson et al., 2011, 2012; Readings et al., 2011). That half of the families in this study had begun disclosing by age 5 of the child is in line with findings from Sweden, where 61% of identity-release donation families had disclosed by the time the child was aged 7 –8 (Lampic et al., 2021).

Whether the remaining 36.1% of mothers in the present study who intended to tell but had not yet done so follow through on their intentions remains to be seen. This is particularly pertinent given that over half of these mothers intended to tell after the child had reached 7 years of age, or otherwise had no clear strategy for when to begin the disclosure process. Parents have previously reported feeling that they had left it too late to disclose when they had not done so by the time their child was aged 6 (Cook et al., 1995). Further evidence from families created using anonymous gamete donation suggests better outcomes for parents and children when disclosure is undertaken before the age of 7 (Ilioi et al., 2017). Whether or not these findings will generalize to families created using identity-release egg donation is worthy of further investigation.

The present study also found that the vast majority of mothers planned to tell their child that they would be able to access the donor*’*s identifying information in the future, with a small number having already begun the process at age 5. This minority of mothers could be seen to be embracing a *‘*seed-planting*’* strategy, whereby details of their conception are shared with the child bit by bit from an early age (Mac Dougall et al., 2007). Whether this approach is adopted by the majority of identity-release egg donation mothers, or whether they otherwise adopt a *‘*right-time*’* strategy whereby parents wait until children are a certain age before sharing these details of their conception, remains to be seen (*Indeku et al., 2013*).

Although planning to disclose their use of egg donation, a handful of mothers were uncertain about whether they would tell their child that they could request the donor*’*s identifying information in the future, over and above informing them that they were donor conceived. Around half of participants in Isaksson and colleagues*’* survey of parents via identity-release sperm donation regarded it to be in their child*’*s best interest to be able to gain access to the donor*’*s identity in the future, although it is unknown whether this translated to actual disclosure (Isaksson et al., 2011). The present study adds to the literature by showing that sharing details of identity release is likely to be part of the disclosure process for the majority of egg donation families in the UK.

It has been suggested that the potential for future donor – child contact implicit in identity-release egg donation may pose a unique threat to mothers who have conceived via egg donation, and that the possibility of donor–child contact may discourage some parents from disclosing their use of donor gametes to their children (Imrie et al., 2020). The present study confirmed the presence of this threat to a certain degree, with a notable proportion of mothers perceiving at least some threat from the possibility of future donor–child contact. These results are in line with the findings of investigations of parental attitudes towards identity-release donation in families with children conceived via sperm donation (Isaksson et al., 2016; Widbom et al., 2021). It is interesting to note that all mothers who understood the implications of identity-release egg donation had either begun to, or planned to, tell their child about how they were conceived; this is noteworthy given the not insubstantial level of threat that some of these mothers perceived from the prospect of future donor child contact.

Although the literature on disclosure among cisheterosexual-couple parents with children conceived via anonymous sperm donation or genetic surrogacy also provides some evidence of perceived threat (Freeman et al., 2016; Jadva et al., 2012; Scheib et al., 2003), correlational analyses in the present study confirmed that perceived donor threat was unrelated to parents*’* disclosure practices. This is perhaps unsurprising given that all of the mothers in the *not disclosed* group intended to disclose in the future.

It is worth noting that a crucial difference between sperm donation fathers, genetic surrogacy mothers and egg donation mothers is that egg donation mothers are provided with the opportunity for gestational bonding, which may reduce the extent to which the donor is perceived as threatening. Indeed, pregnancy has been identified as an important period for gestational mothers who lack a genetic connection with their child, and has been described as a way of achieving biological equality with their partner (who does have a genetic relationship to their child), thus solidifying their sense of legitimacy of and security in their role as parent (Becker, 2000; Finkler, 2000; Nordqvist, 2017; Shaw et al., 2023). It is therefore possible that the gestational relationship of egg donation mothers with their children to some degree explains the lack of association between donor threat and disclosure. However, several studies have found that, although important, pregnancy alone is not sufficient for making egg donation mothers feel secure in their role as mother (Imrie et al., 2020; Kirkman, 2008; Lysons et al., 2022).

An alternative explanation for the lack of association between donor threat and disclosure status is that, as many of the mothers in this sample were advised by their clinic to disclose to their child in their early years or had otherwise come to this conclusion during their own research, it is possible that they considered early disclosure the officially sanctioned and, therefore, correct course of action, despite their own feelings about the prospect. It is likely that the increasing prevalence of direct-to-consumer genetic testing, and the subsequent increasing risk of accidental discovery of one*’*s donor conception, is further contributing to clinics*’* advice to parents to disclose (Flynn, 2022; Harper et al., 2016). As Freeman notes, the introduction of a donor identification system automatically ascribes significance to the genetic link between donor and child (Freeman, 2015). This legislative change has been viewed by some as the further geneticization of the family, and may compound the belief in some donor conception parents that genetic identity, i.e. a knowledge of one*’*s genetic origins, is more crucial for optimal personal identity development than other, more socially embedded forms of identity (Brown and Wade, 2022; Lysons et al., 2022; Turkmendag, 2012).

Conversely, among those mothers who did not understand the implications of identity-release donation, almost half were either undecided about whether to, or planned not to, disclose their use of egg donation to their child. All of the mothers who were unsure whether to, or planned not to, tell were in this group. It is possible that the co-occurrence of these mothers*’* lack of understanding of identity-release donation, and their disinclination to disclose their use of egg donation, reflects a subsample of mothers who are less able to accept that they had had to use donor eggs to conceive. Some donor conception parents have been found to deliberately disengage from donor information to manage the psychological and emotional load of having used donor gametes (Widbom et al., 2021; Zadeh et al., 2016), and to facilitate the feeling of being able to fully own the identity of being the child*’*s parent (Imrie et al., 2020). It is therefore possible that these mothers were, perhaps unconsciously, participating in a pattern of defensive denial that Konrad (2005) describes as an *‘*active not knowing*’*, although further research into these mothers*’* motivations for non-disclosure will be necessary to answer this question directly.

A notable strength of this study is that it is the first to quantify donor threat in order to explore it in relation to other family process variables among a clinic-recruited, and therefore representative, sample of identity-release egg donation families. A limitation of the study was that 77% of participants who were uncontactable, or declined to take part at phase two, had planned not to disclose, or were uncertain about disclosure to their child (Lysons et al., 2022). It is, therefore, possible that the present sample over-represents participants who favoured disclosure.

A further limitation of the present study is that the vast majority of the sample identified their ethnicity as White British, thus limiting the generalizability of the present study*’*s findings to non-British and non-white British individuals. Census data suggest that non-white British couples and individuals find it harder to access fertility treatment (HFEA, 2021b), with similar patterns in the USA (Armstrong and Plowder, 2012). Furthermore, cultural and religious factors have been found to influence assisted reproduction usage throughout much of Europe (*Preag and Mills, 2017*) and Southwest Asia (Ali et al., 2011; Senol et al., 2019; Serour and Serour, 2021). Although few empirical data exist on disclosure attitudes and rates among ethnic minority groups in the UK and beyond, the limited literature suggests that the use of third-party reproduction is highly stigmatized, and that couples closely manage information sharing regarding their use of donor gametes (Blell, 2018; Culley et al., 2013; Hudson and Culley, 2013). Further research into the attitudes towards disclosure among parents from ethnic minority backgrounds is therefore necessary.

Given that egg donation fathers share a genetic link with their child and given that, regardless of donation type, mothers tend to be more likely to take responsibility for disclosure in donor gamete families (Blake et al., 2010; Lycett et al., 2005; Paul and Berger, 2007), the present findings regarding donor threat and disclosure may not apply to egg donation fathers. Low paternal participation rates are a well-documented issue in family psychology research, with systematic reviews consistently demonstrating much higher recruitment and retention rates among mothers than fathers (Davidson et al., 2016; Phares et al., 2005). Future research into families created via identity-release egg donation should focus on fathers*’* perspectives, in order to examine how they understand the genetic asymmetry within their families, and how this relates to their feelings about donor threat and disclosure to their child.

Nonetheless, the findings provide important insights into mothers*’* disclosure practices. That mothers are disclosing in spite of sometimes pronounced levels of perceived donor threat is of relevance to policy and practice regarding identity-release donation. The present study also found that, where data were available from both mothers and fathers, most couples (77.1%) agreed about whether or not to disclose to their child that they were donor conceived. This is in line with findings from a Swedish survey of 111 heterosexual-couple parents of children conceived via identity-release egg and sperm donation, which found that 76% of respondents agreed with their partner about their disclosure strategy (Isaksson et al., 2012).

Isaksson and colleagues also found that disagreement about disclosure strategy was significantly associated with poorer level of relationship satisfaction between couples. This is particularly pertinent given findings that indicate better family functioning among donor conception families where disclosure has been undertaken by the parents jointly (Paul and Berger, 2007). Further therapeutic support, such as one-to-one and couples*’* counselling sessions or group workshops, should therefore provide parents with the opportunity to explore their feelings about their use of identifiable egg donation, to address any tensions between feelings of threat and a desire to disclose, and to help arrive at a mutually satisfying disclosure strategy.

## Figures and Tables

**Table 1:** Identity-Release Egg Donation Mothers’ Disclosure Intensions At Phase One, and Disclosure Practices At Phase Two By Level Of Understanding About Identity-Release Donation

Disclosure decision	Phase one (*n* =73)	Phase two (*n* =61)		
		Total (n = 61)	Mothers who do not understand identity-release donation (*n* = 17)	Mothers who understand identity-release donation (*n* = 44)
Disclosure about egg donation, *n* (%)				
Started telling	–	31 (50.8)	4 (23.5)	27 (61.4)
Plansto tell	55 (75.3)	22 (36.1)	5 (29.4)	17 (38.6)
Uncertain	12(16.4)	4 (6.6)	4(23.5)	–
Plans not to tell	6(8.2)	4 (6.6)	4(23.5)	–
Disclosure about identity-release, *n* (%)				
Started telling	–		–	4 (9.1)
Plans to tell	–		–	37 (84.1)
Uncertain	–		–	3 (6.8)

**Table 2:** The Age At Which Mothers Planned To Tell Their Children That They Were Donor Conceived, Reported At Phase Two

Planned age of disclosure	Total (*n* =22)
Before the age of7, *n* (%)	9 (40.9)
Between 7 and 10 years, *n* (%)	4 (18.2)
During the child’s teens, *n* (%)	3(13.6)
Uncertain, *n* (%)	6 (27.3)

**Table 3:** Agreement In Disclosure Strategy Between The Parents At Phase Two

Level of agreement	Couples (total *n* = 48)
Agreement between couples, *n* (%)	37(77.1)
Disagreement between couples, *n* (%)	11 (22.9)
Types of mismatch, *n* (%)	
Mother started telling, father planning to tell	4 (36.4)
Mother planning to tell, father started telling	3 (27.3)
Mother uncertain, father planning not to tell	2(18.2)
Mother planning to tell, father planning not to tell	1(9.1)
Mother planning not to tell, father planning to tell	1 (9.1)

## Data Availability

The data that has been used is confidential.

## Key Message

Most mothers of infants conceived via identity-release egg donation intended to tell their children how they were conceived; half had disclosed by 5 years. Despite some mothers perceiving future donor–child contact as threatening, most intended to inform their child of their right to access donor-identifying information at age 18.
